# Georgia State Dental Society

**Published:** 1873-06

**Authors:** M. S. Jobson

**Affiliations:** Corresponding Secretary.


AMERICAN JOURNAL

OF

DENTAL SCIENCE.

Vol. VII. THIRD SERIES—JUNE, 1873. No. 2.

### ARTICLE I.

### *Georgia State Dental Society.*

The regular annual meeting of this body assembled in the skating hall of the Rankin House in Columbus, April 2nd, 1873, at 10 o'clock A. M., Dr. E. M. Allen, of Marrietta, President, in the chair.

Drs. W. J. Fogle, W. T. Poole and T. W. Hentz, of Columbus, and J. L. Fogg, of Barnesville, were elected to active membership. Dr. Ford, of the committee on operative dentistry, made report and spoke particularly of the Morrison dental engine, mentioning the important points of its greatly facilitating operations, and giving less pain to the patient. He recommended the machine to all. Drs. Holland and Lowrance spoke of it in the highest terms.

Dr. Parsons enquired how members fill molar crown cavities where the nerve is nearly exposed.

Dr. Campbell, of Barnesville, used Hill's stopping on the floor of the cavity dipped in carbolic acid or creasote, and had more success than in any other way.

Dr. Ford made statement of his filling a first inferior molar on the grinding surface, as follows: Excavated thoroughly with the Morrison engine, except leaving a small portion of partially decayed dentine over the pulp, flowed the cavity with creasote, and covered its floor with bibulous paper, about three thicknesses, saturated with a solution of pure rubber dissolved in chloroform, after allowing the chloroform to evaporate, filled the cavity with cement Plombe; there was no pain caused upon the introduction of the filling.

Dr. Fogle used Hill's stopping saturated with chloralum and creasote.

Dr. Tigner asked what the physiological action of creasote was.

Dr. Parsons said it contracted the blood vessels, and relieved the pulp of its engorgements.

Dr. Tigner desired to hear the remarks or experience of members upon the use and application of the rubber dam. Treatment of teeth where the pulps are exposed. Capping and devitalizing pulps—which the surer practice.

Dr. Ford herewith expressed himself in glowing terms with regard to the great utility of the rubber dam. It could, he said, with care and perseverance, be applied to any tooth, and was of incalculable service, and to him almost indispensable for the performance of perfect, especially if extended operations. In cases of difficulty of application, such as upon a conically shaped lower molar, he used the Spankler rubber clamp by first pressing it through the rubber, then placing the clamp well down upon the neck of the tooth and forcing the rubber below it with a burnisher. In cases of extreme hypersensitive dentine, he found great advantage in applying the rubber and keeping the cavity dry while excavating.

Dr. McElhaney considered it one of the greatest advantages yet gained in operative dentistry, and thought Dr. Barnum merited the thanks of the whole profession. A motion was made and adopted that a clinic be had at the office of Dr. Fogle for the special purpose of demonstrating its different modes of application for the benefit of those who have heretofore been unsuccessful in its application. Dr. Ford was requested to act as demonstrator. At the clinic the most difficult application was to a right inferior third molar which was both somewhat retroverted and inclined lingually and conical in form, the cervical portion being the largest; after perforating the rubber he passed the claws or beaks of a Spankler clamp through—took hold of the clamp with a small pair of forceps—folding the rubber back over them, forced the clamp down in position, and then with a burnisher worked the rubber below the beaks of the clamp. The application was perfect and demonstrated the great utility of the rubber dam in difficult cases.

As to treatment of exposed nerves, Dr. Ford had followed the practice of capping with cement Plombe for years, and had been so successful found no necessity for a change. He vitalized pulps in comparatively few cases—treated front teeth successfully that had been ulcerated for years.

Dr. Tigner had capped nerves but had been unsuccessful —thought it better to devitalize and remove; he used creaote freely and had been very successful.

Dr. McDonald had capped nerves successfully with lead.

The discussion then turned upon ulcerated teeth, wherein Dr. Parsons remarked, pus in the canal of a tooth cannot pass through the foramen unless forced, but gases generated by decomposition may and produce inflammation in the surrounding parts. Unhealthy pus was decomposition of the pulp and is dark, while healthy pus was white.

Dr. Holland agreed with Dr. Parsons, but could not accept the term "healthy pus."

As to gravitation of pus of which Dr. Tigner had spoken, Dr. Ford said it would no sooner gravitate downward than upward, but would make its exit through the orifice which by nature was most practicable.

Dr. Fogle said the more important point for us to discuss, is how to get rid of the puss after its formation.

Dr. Ford had seen teeth replaced with success after the necrosed apex had been excised.

Dr. Parsons made the following remarks: He claimed to have discovered a nervous property in the article gaultheria or wintergreen, which when properly used, is a good substitute for common anæsthetics. He uses it in the form of essence,—four ounces of the oil to one quart, ninety-five per cent. alcohol—applies a little on a napkin, directs the patient to inhale through the nose, and continue until the scalp becomes blushed. Consciousness is not at all suspended, but nervous sympathy controlled so that teeth may be extracted with very little or no shock to the nervous system. He did not claim that no local pain was felt, but that by its use, the most nervous person is abundantly able to bear extraction. It is also excitant for bathing the external parts affected in cases of facial neuralgia.

Dr. Ford spoke at some length on the endowment of one of the Southern dental colleges.

Dr. Poole expressed a willingness to contribute annually for ten years if an endowment was decided upon.

Dr. Shackelford also expressed a willingness to contribute annually.

Dr. Parsons thought it best to give as much as we could all at once, that the faculty could not depend upon subscriptions—the amount raised should be $150,000 or $200,000.

Dr. Ford said it was the purpose to send a circular to each Southern dentist in relation to the endowment.

After discussion of some length, Dr. Parsons offered the following, which was unanimously adopted.

*Resolved*, that this Society heartily endorse the action of the Southern Dental Association in raising an endowment fund to endow a Southern dental college, and will use our best efforts to bring the scheme to a practical issue.

Dr. Ford of the committee on legislation, read the Act of the last Legislature regulating the practice of dentistry in the State.

At clinics, Drs. Ford, Holland, McElhaney and Jobson, operated.

The following officers were elected for the ensuing year :

*President.*—A. C. Ford, of Atlanta.

*1st Vice President.*—I. W. Hentz, of Columbus.

*2nd Vice-President.*—G. W. McElhaney, of West Point.

*Corresponding Secretary.*—M. S. Jobson, of Perry.

*Recording Secretary.*—L. D. Carpenter, of Atlanta.

*Treasurer.*—H. A. Lowrance of Athens.

Standing Committees for the ensuing year were appointed as follows:

*Physiology and Dental Histology.*—J. P. H. Brown, of Augusta, and George Paterson, of Waynesboro.

*Dental Pathology and Surgery.*—M. H. Thomas, of Monroe, and E. Parsons, of Savannah.

*Dental Chemistry and Therapeutics.*—F. Y. Clark, of Savannah, and R. I. Hampton, of Rome.

*Operative Dentistry.*—E. M. Allen, of Marietta, and R. A. McDonald, of Griffin.

*Mechanical Dentistry.*—W. J. Fogle, of Columbus, and J. L. Fogg, of Barnesville.

*Dental Education.*—F. Y. Clark, of Savannah, and M. A. Shackelford, of Hogansville.

*Clinical Committee.*—R. A. McDonald, of Griffin; M. S. Jobson, of Perry; W. I. Poole, of Columbus; and H. A. Lowrance, of Athens.

*Committee of Arrangements.*—Samuel Hape, J. I. Campbell, and L. D. Carpenter, of Atlanta.

A new Constitution and By-Laws were adopted which fixes Atlanta as the regular place of future meetings

M. S. Jobson, *Corresponding Secretary.*

